# Risk for imbalanced blood pressure pattern among incarcerated women: Middle-Range Theory

**DOI:** 10.1590/0034-7167-2023-0288

**Published:** 2024-07-15

**Authors:** Gabrielle Pessôa da Silva, Camila Takáo Lopes, Marcos Venícios de Oliveira Lopes, Ryanne Carolynne Marques Gomes Mendes, Jaqueline Galdino Albuquerque Perrelli, Lívia Maia Pascoal, Suzana de Oliveira Mangueira, Francisca Márcia Pereira Linhares

**Affiliations:** I Universidade Federal de Pernambuco. Recife, Pernambuco, Brazil; II Universidade Federal de São Paulo. São Paulo, São Paulo, Brazil; III Universidade Federal do Ceará. Fortaleza, Ceará, Brazil; IV Universidade Federal do Maranhão. São Luís, Maranhão, Brazil

**Keywords:** Nursing Diagnosis, Nursing Theory, Arterial Pressure, Women, Prisons, Diagnóstico de Enfermería, Teoría de Enfermería, Presión Arterial, Mujeres, Prisiones

## Abstract

**Objectives:**

to develop a Middle-Range Theory for the Risk for imbalanced blood pressure pattern among incarcerated women.

**Methods:**

theoretical development study to obtain the theoretical-causal validity of the Nursing Diagnosis Risk for unstable blood pressure. The Middle-Range Theory was developed according to six stages: establishment of the approach to developing the theory; definition of the conceptual models to be later analyzed; definition of the main conceptions; a pictorial diagram; propositions; causal relationships and evidence for practice.

**Results:**

two attributes and 20 antecedents related to imbalanced blood pressure were identified, a pictorial diagram was developed, and nine theoretical propositions were presented.

**Conclusions:**

the theory developed here favors the diagnostic reasoning of nurses and contributes to planning actions to promote the cardiovascular health of incarcerated women. A new proposition for the diagnosis of Risk for unstable blood pressure was also structured with a new title, definition, and etiological factors.

## RESUMEN

**Objetivos:**

desarrollar una Teoría de Alcance Mediano para Riesgo de patrón de presión arterial desequilibrada, en mujeres encarceladas.

**Métodos:**

desarrollo teórico para la validez teórica-causal del Diagnóstico de Enfermería Riesgo de Presión Arterial Inestable. Fue construida una Teoría de Alcance Mediano en seis etapas: definición del abordaje para construcción de la teoría; definición de modelos conceptuales que serán analizados; definición de conceptos principales; desarrollo del diagrama pictórico; construcción de proposiciones; establecimiento de relaciones causales; y, evidencias para la práctica.

**Resultados:**

fueron identificados dos atributos y 20 antecedentes relacionados al patrón de presión arterial desequilibrado; se construyó diagrama pictórico; se presentaron nueve proposiciones teóricas.

**Conclusiones:**

la teoría desarrollada favorece el raciocinio diagnóstico de enfermeros y contribuye para planificar acciones promotoras de salud cardiovascular en mujeres encarceladas. Fue posible estructurar nueva propuesta para el diagnóstico Riesgo de Presión Arterial Inestable, con nuevo título, nueva definición y nuevos factores etiológicos.

## INTRODUCTION

Crowds, noise, and conflicting and threatening relationships in a context of incarceration, smoking, alcohol consumption, and illicit drug use^(1)^ promote a stressful environment, which contributes to elevated blood pressure and increases the number of systemic arterial hypertension (SAH) cases even after individuals are released from prison^(2)^.

A prevalence of 24.2% of incarcerated hypertensive women is found in Brazil, mostly young women (33 years old on average)^(3)^. Incarcerated women are generally more affected than men and have difficulty maintaining normal blood pressure levels due to inadequate diet and sedentary lifestyle, as opportunities to exercise within a penal institution are minimal^(4)^.

Hence, nurses providing care in penal institutions should identify the risk factors for alterations in blood pressure to prevent cardiovascular events^(5)^. NANDA International Nursing Diagnoses (NANDA-I) can facilitate the identification of these factors, though inconsistencies in the diagnostic structure may compromise identifying and naming such a phenomenon^(6)^.

Risk for unstable blood pressure is a nursing diagnosis (ND) defined in NANDA-I as “vulnerable to fluctuations in blood pressure that may compromise health”^(6)^. This unclear definition may impair a nurse’s diagnostic reasoning and clinical judgment. Furthermore, NANDA-I presents only two risk factors for this ND, i.e., inconsistent compliance with the pharmacological regimen and orthostasis, which may hinder nurses from recognizing such a phenomenon.

Studies are needed to support the validity evidence of an ND to expand its use in different populations. In this context, the Middle-Range Theory (MRT) is the preferred methodological approach for obtaining the theoretical-causal validity of a diagnosis^(7)^.

As the ND Risk for unstable blood pressure requires a clear definition and more risk factors that enable precise identification, obtaining its theoretical-causal validity by applying MRT is recommended. No scientific studies building a nursing theory for this phenomenon were found. Hence, such a study is needed to contribute to the operationalization of the Nursing Process when care is provided to incarcerated women. For this reason, this study represents an innovation in the nursing field.

## OBJECTIVES

To develop an MRT for the Risk for imbalanced blood pressure pattern among incarcerated women.

## METHODS

### Ethical aspects

There was no need to ask for the Institutional Review Board’s approval as this is a theoretical development study and does not require the direct participation of human subjects.

### Study design

This theoretical development study aimed to obtain the theoretical-causal validity of the ND Risk for unstable blood pressure^(7)^. Thus, an MRT was developed based on an integrative literature review and the concepts of the Roy Adaptation Model (RAM)^(8)^.

The theory’s development followed the six steps proposed by Lopes, Silva, and Herdman (2017), which reiterate Roy’s (2014) framework: establishment of the approach to developing the theory; establishment of the conceptual models to be later analyzed; definition of the main concepts; pictorial diagram; propositions; causal relationships and evidence for practice^(9-10)^. At the end of the theoretical development, we propose to refine the ND addressed here with a new title, which gave the name to the MRT developed in this study.

### Establishing the approach to develop the Middle-Range Theory and the conceptual models

An integrative literature review and the RAM concepts^(8)^ were the approaches adopted to develop the MRT. This model was chosen because it considers that the alterations incarcerated women experience upon entering prison directly interfere with various health aspects, including cardiovascular health. The reason is that a prison is an overcrowded environment, where there is a high level of stress, poor access to the health system, and few opportunities to change lifestyle^(1)^. These aspects demand women to adapt their way of life, which implies relevant repercussions for their blood pressure.

Roy’s concepts adopted here are those of focal, contextual, and residual stimuli^(8)^. Focal stimuli are the closest to individuals and cause the most significant impact on change. Internal and external contextual stimuli influence the focal stimulus. Residual stimuli have non-central effects^(8)^ and concern imbalanced blood pressure’s clinical antecedents (risk factors, associated conditions, and at-risk populations). Finally, in this study, the adaptive problem is the imbalanced blood pressure pattern, which was considered a potential behavior among incarcerated women due to the clinical history/stimuli identified here. According to RAM, this behavior is classified under a physiological model^(8)^.

### Main concepts

The main concepts adopted here were essential attributes and clinical history^(9)^ of Risk for imbalanced blood pressure. Essential attributes are the ND’s conceptual core characteristics. The clinical antecedents for risk diagnoses can be risk factors (elements that increase one’s vulnerability to the diagnosis and are subject to autonomous nursing interventions), at-risk populations (non-modifiable demographic characteristics and health history), and associated conditions (medical diagnoses, medical devices, and medicines)^(6,9)^. These concepts were identified in an integrative literature review, according to the following: problem identification, literature search, data evaluation and analysis, and presentation of results^(11)^.

### Problem identification

The problem was operationalized through PICo - P (Population): incarcerated women; I (Interest): essential attributes and clinical history; and Co (Context): female incarceration. Hence, the following research question emerged: “What are the essential attributes and clinical antecedents of risk for imbalanced blood pressure among incarcerated women?”.

### Literature review

The bibliographic search was conducted in May 2021, using the MeSH terms “arterial pressure,” “prisons,” “prisoners,” “risk factors,” and “women” in the Scopus, CINAHL, Medline/PubMed, Web of Science, Embase, Science Direct, and Cochrane databases. The Health Sciences Descriptors (DeCS) “blood pressure,” “hypertension,” “prisons,” and “women” and their equivalent in Portuguese and Spanish were adopted for the LILACS database. No timeframe or language restrictions were applied. The following search strategies were adopted.

1) Scopus, CINAHL, Medline/PubMed, Web of Science, Embase, Science Direct, and Cochrane: “arterial pressure” AND (prisons OR prisoners) AND “risk factors” AND women

2) LILACS: #1) Hypertension AND Women AND Prisons; #2) “Arterial pressure” AND Women AND Prisons; #3) *Hipertensão* AND *mulheres* AND *prisões*; #4) *Pressão arterial* AND *Mulheres* AND *Prisões*; #5) *Hipertensión* AND *Mujeres* AND *Prisión*.

### Data evaluation

Two researchers independently read the studies’ titles and abstracts and the full texts, collecting the following information: author(s), year of publication, study design, country of origin, essential attributes, and clinical history of imbalanced blood pressure. Inclusion criteria were primary observational studies or literature reviews that defined the study phenomenon and/or described factors contributing to the imbalanced blood pressure pattern among incarcerated women. Exclusion criteria were editorials, annals of events, and letters to the editor.

### Data analysis

The studies’ evidence levels were assessed according to the classification: Level 1: experimental studies, Level 2: quasi-experimental studies, Level 3: observational analytical studies, Level 4: observational descriptive studies, and Level 5: expert opinions and research bench. Methodological, qualitative, cross-sectional analytical studies or literature reviews that were not systematic were classified at level 5^(12)^.

### Presentation of results

The results were categorized as essential attributes or clinical history. Clinical antecedents were categorized as focal, contextual, or residual stimuli based on Roy’s (2009) theoretical assumptions about the RAM^(8)^ concepts, the causal relationships presented in the literature, and our reflections based on practical experiences.

A total of 2026 studies were found. After removing duplicates, 755 remained for the reading of titles and abstracts. The full texts of 36 studies were read, and 25 were included in the final sample. The data from the selected studies are summarized in Chart 1.

**Chart 1:** Studies included in the Literature Review to develop the Middle-Range Theory of Risk for imbalanced blood pressure among incarcerated women, Recife, Pernambuco, Brazil, 2023

Authors, year, country of origin	Study design and evidence level	No. of attributes extracted	No. of CA extracted
Bondolfi et al., 2020^(2)^ United Kingdom	Systematic review Level 1	1	7
Nolan e Stewart, 2017^(14)^ Canada	Cross-sectional study Level 4	1	8
Agyapong et al., 2017^(15)^ United Kingdom	Systematic review Level 1	1	9
Arries and Maposa, 2013^(16)^ USA	Integrative Review Level 5	2	11
Bautista-Arredondo et al., 2015^(17)^ Mexico	Cross-sectional study Level 4	2	8
Galvão et al., 2019^(18)^ Brazil	Cross-sectional study Level 4	1	7
Hachbardt et al., 2020^(19)^ Brazil	Cross-sectional study Level 4	1	12
Khavjou et al., 2017^(20)^ USA	Cross-sectional study Level 4	1	9
Lagarrigue et al., 2017^(21)^ France	Cross-sectional study Level 4	1	6
Plugge et al., 2009^(22)^ England	Descriptive prospective study Level 4	1	8
Silva et al., 2021^(23)^ Brazil	Methodological study Level 5	1	11
Vera-Remartínez et al., 2018^(24)^ Spain	Cross-sectional study Level 4	1	11
Wildeman et al., 2013^(25)^ USA	Cross-sectional study Level 4	1	8
Udo, 2019^(26)^ USA	Cross-sectional study Level 4	1	9
Nara and Igarashi, 1998^(27)^ Japan	Cross-sectional study Level 4	1	5
Gebremariam et al., 2018^(28)^ United Kingdom	Systematic review Level 1	--	3
Herbert et al., 2012^(29)^ United Kingdom	Systematic review Level 1	--	3
Nucci et al., 2019^(30)^ Italy	Methodological study Level 5	--	4
Mohan et al., 2018^(31)^ Holland	Systematic Review Level 1	--	4
Wangmo et al., 2018^(32)^ Switzerland	Qualitative study Level 5	--	2
Ritter et al., 2011^(33)^ USA	Systematic review Level 1	--	1
Valera et al., 2016^(34)^ USA	Qualitative study Level 5	--	1
Gilles et al., 2008^(35)^ Australia	Cross-sectional study Level 4	--	3
Johnson et al., 2019^(36)^ Canada	Cohort Level 3	--	5
Fazel and Baillargeon, 2011^(37)^ United Kingdom	Integrative Review Level 5	--	3

*CA - Clinical Antecedents.*

### Pictorial diagram, propositions, causal relationships, and evidence for practice

A graphical summary was designed for the interrelationships between the concepts to summarize the MRT’s elements. The propositions were developed based on explicit statements to show the relationship between clinical history and diagnosis, highlighting their specificities. The causal relationships between clinical antecedents and imbalanced blood pressure were also identified and described based on integrative reviews and other scientific articles when the former did not present such relationships. This last stage presents the clinical relationships that lead to diagnostic reasoning in nursing^(9)^.

Considering what is provided for in the ISO 18104 standard about nursing reference terminologies^(13)^, we propose to refine the title, definition, and etiological factors of the ND Risk for unstable blood pressure based on the essential attributes, antecedents identified in the literature review, and the causal relationships established in the MRT. The NANDA-I definitions regarding the diagnostic title were considered, with the diagnostic status (Risk diagnosis) associated with the focus and judgment axes. The definition was developed to provide a clear and accurate description of the diagnostic title. Risk factors were considered as antecedents that increase an individual’s vulnerability to undesirable human responses, the associated conditions are related to diagnoses/procedures/medical devices, and the populations at risk are groups of individuals with greater vulnerability to the phenomenon^(6)^.

## RESULTS

### Main concepts

Two essential attributes were identified in the studies as main concepts, which were the “recurrent elevation of the force of the blood against the arterial wall above the desired level”^(2,14-26)^ and the “recurrent decrease in the force of blood against the arterial wall below the desired level”^(16-17,27)^. Twenty clinical antecedents related to the imbalanced blood pressure pattern were also identified and categorized according to the RAM; descriptions are presented in Chart 2.

**Chart 2:** Categorization of the clinical antecedents of the Risk for imbalanced blood pressure pattern among incarcerated women, according to Roy Adaptation Model’s focal, contextual, and residual stimuli, Recife, Pernambuco, Brazil, 2023

Stimuli characterization	Stimuli definition	Clinical antecedents
Focal Stimuli	Focal stimuli directly affect incarcerated women and are mainly responsible for increasing their vulnerability to developing an imbalanced blood pressure pattern^(8)^.	Sedentary lifestyle^(2,15-24,27-31)^ Hyper caloric diet^(15-16,18-20,22-24,28-31)^ High sodium diet^(2,15,18-20,22-24,28-32)^ Smoking^(2,14-27,30-31,33-36)^ Harmful use of illicit substances^(13,15,17,25,27,35-37)^ Harmful consumption of alcohol^(14-15,18-19,22-23,25-27,35-37)^ Anxiety^(21,23,25)^ Stress^(2,15-16,19,23,26)^ Post-Traumatic Stress Disorder^(16)^ Metabolic syndrome^(16,21,2 4)^
Contextual stimuli	Contextual stimuli indirectly influence incarcerated women to develop an imbalanced blood pressure pattern^(8)^.	Insomnia^(15,19)^ Excess body weight^(2,14-30)^ Dyslipidemia^(2,14-17,20-21,23-24,26-27)^ Cardiovascular disease^(14,16,19-20,25-26)^ Diabetes^(19,2 6)^
Residual stimuli	Residual stimuli less intensely promote imbalanced blood pressure patterns in incarcerated women. It is not completely clear what role these stimuli play in the occurrence of an imbalanced blood pressure pattern^(8)^.	Insufficient knowledge/understanding of risk factors^(16,23)^ Individuals with a family history of hypertension^(19)^ Individuals in social vulnerability^(2,16-20,22,24-26,31,36)^ Women^(14,16-17,24,26,31,37)^ Individuals over 30 years of age^(14-20,22,25-26)^

### Pictorial diagram

**Figure 1:**
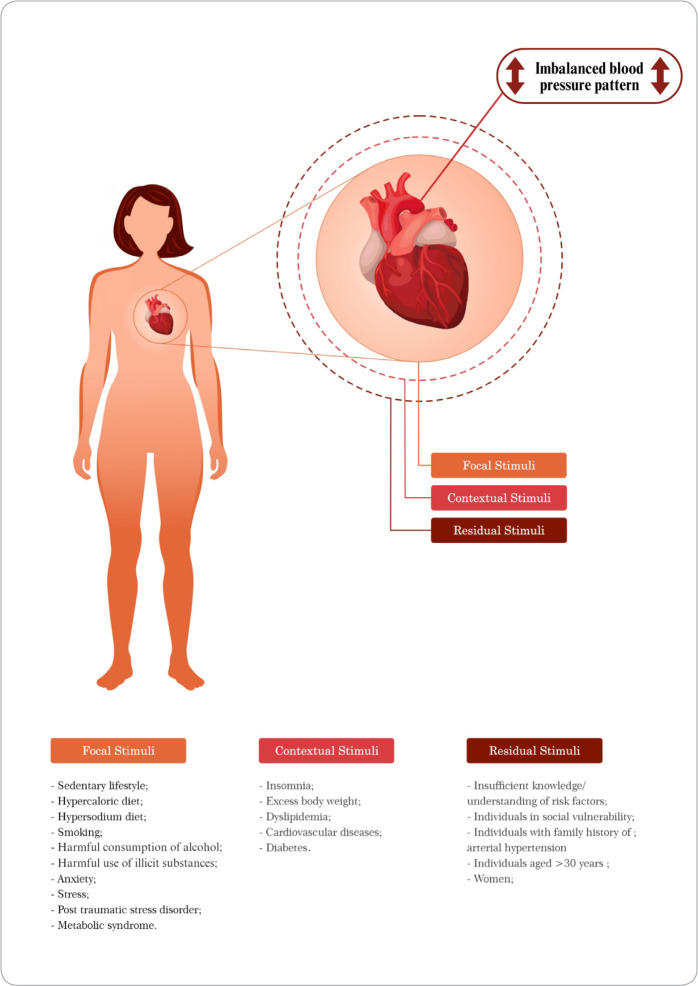
Pictorial diagram of Risk for imbalanced blood pressure pattern, considering the Roy Adaptation Model, Recife, Pernambuco, Brazil, 2023

The pictogram, presented in Figure 1, demonstrates the interrelationships between the concepts that involve Risk for imbalanced blood pressure pattern among incarcerated women. The heart is highlighted to represent the human organism (incarcerated woman). The human heart is affected by various stimuli that may lead to imbalanced blood pressure pattern, such as recurrent elevation or decrease in the force of blood against the arterial wall, above or below desirable levels, represented by the exit of blood from the aorta artery. The dashed circles indicate that the stimuli can be reclassified depending on the intensity they affect people.

### The Middle-Range Theory’s propositions of Risk for imbalanced blood pressure pattern among incarcerated women

Nine theoretical propositions emerged, based on the interrelationship between the concepts (essential attributes and clinical antecedents/stimuli) of Risk for imbalanced blood pressure pattern among incarcerated women, based on the RAM:

1. Focal, contextual, and residual stimuli affect a prisoner’s physiological mode and compromise cardiovascular functioning, increasing her vulnerability to developing imbalanced blood pressure ineffective behavior.
2. Focal, contextual, and residual stimuli are intrinsic or extrinsic to incarcerated women. They may have been acquired before imprisonment and persist in prison, or they may have started only after incarceration.
3. Focal stimuli include a sedentary lifestyle, high-calorie diet, high-sodium diet, smoking, and the harmful consumption of alcohol and illicit substance use. Incarcerated women frequently adopt these lifestyle habits, which directly affect blood pressure, resulting in a imbalanced blood pressure pattern (elevation or decrease).
4. Focal stimuli include anxiety, stress, and Post-Traumatic Stress Disorder, which are related to confinement in prison and trauma experienced before or during imprisonment, directly influencing the development of unstable blood pressure behavior (elevation) among incarcerated women.
5. Metabolic syndrome is a focal stimulus that may result from the accumulation of some cardiovascular risk factors in incarcerated women and strongly favors imbalanced blood pressure pattern (elevation), further increasing these women’s vulnerability.
6. Insomnia is a contextual stimulus that enhances imbalanced blood pressure pattern (elevation) and may be related to the physical structure of prisons, in addition to the mental health of incarcerated women.
7. The contextual stimuli concerning excess body weight, dyslipidemia, cardiovascular disease, and diabetes are clinical conditions that enhance imbalanced blood pressure pattern (elevation) among incarcerated women.
8. Insufficient knowledge/understanding of risk factors is a residual stimulus that affects self-care to maintain healthy blood pressure; hence, it indirectly influences imbalanced blood pressure pattern (elevation) among incarcerated women.
9. Residual stimuli concern individuals with a family history of high blood pressure, experiencing social vulnerability, aged over 30, and being a woman. These stimuli less intensively influence the development of imbalanced blood pressure pattern (elevation).

### Proposition of the Nursing Diagnosis Risk for unstable blood pressure

Thus, based on the MRT developed here and its elements, we present a new proposition for the ND Risk for unstable blood pressure to help clarify and refine its structure. This proposition is based on the theoretical-causal validity process of the ND Risk for unstable blood pressure performed through this MRT. Its structure is presented below:

Title: Risk for imbalanced blood pressure pattern; Definition: vulnerability to recurrent increase or decrease in the force exerted by the blood on the artery wall, above or below the desired level, which can compromise health; Risk factors: insufficient knowledge/understanding of risk factors, excess body weight, sedentary lifestyle, high-calorie diet, high-sodium diet, metabolic syndrome, smoking, harmful use of illicit substances, harmful consumption of alcohol, anxiety, stress, and insomnia; Associated conditions: dyslipidemia, diabetes, Post-Traumatic Stress Disorder, and cardiovascular disease; Populations at risk: individuals with a family history of high blood pressure, socially vulnerable individuals, women, over 30 years of age.

The title change was proposed to more clearly represent the essential attributes of the phenomenon under study, facilitating nurses to recognize this ND during clinical judgment and diagnostic reasoning. The essential attributes “Recurrent increase in the force of blood against the arterial wall above the ideal parameter” and “Recurrent decrease in the force of blood against the arterial wall below the ideal parameter” supported the title reformulation. Thus, the terms “pressure pattern” and “imbalanced” were adopted because they better portray blood pressure levels that can compromise health since instability is a characteristic of blood pressure, which may occur throughout the day due to various causes and mechanisms, without necessarily compromising one’s health. However, an imbalanced blood pressure pattern recurrently above or below desirable levels can compromise an individual’s health.

Changes are also proposed to risk factors, associated conditions, and at-risk populations, i.e., inclusions, and expansions are proposed to the ND presented by NANDA-I. One could assume that such changes would be explained by the specific population addressed when we developed the MRT; however, it is noteworthy that these findings are verified for the general population according to the causal relationships discussed below.

## DISCUSSION

MRTs should encompass less abstract concepts and more closely express the details of nursing practice. The theories for this science must focus on a specific part of a given phenomenon, seeking to include a shorter number of concepts and propositions, which must be related to research and practice^(38)^.

An MRT study in cardiology has recently been developed to address deficient knowledge in individuals with heart failure. It was developed based on the same framework adopted in this study and presented 11 propositions and four causal relationships to guide nurses in their clinical judgment^(39)^.

The MRT of Risk for imbalanced blood pressure pattern was developed here based on an investigation of this phenomenon conducted among incarcerated women. The objective was to obtain a causal explanation in this field of knowledge and guide the nursing process and clinical practice. According to the studies composing the MRT, a recurrent increase or decrease in the force of blood against the arterial wall, above or below ideal levels, can be considered an essential attribute of Risk for imbalanced blood pressure pattern among incarcerated women^(2,14-25,27)^. Elevated blood pressure was the main factor identified here that can compromise the cardiovascular health of these women. Some studies highlight blood pressure parameters above or equal to 130x85 mmHg^(19,21)^, which is similar to the parameter adopted by the American Heart Association in 2018^(40)^, which establishes that blood pressure levels above or equal to 130x80 mmHg should be considered Systemic Arterial Hypertension (SAH).

Other studies^(15,20,22,24)^ adopt the SAH parameters recommended by the Brazilian and European guidelines for arterial hypertension, which are greater than or equal to 140x90 mmHg^(40-42)^. The blood pressure parameters established worldwide reinforce the need to adopt increasingly early measures to prevent high blood pressure.

The studies included in this MRT do not mention a specific parameter for arterial hypotension. However, the individual’s usual blood pressure level must be considered. Usually, blood pressure below 90x60 mmHg is considered hypotension, especially when associated with dizziness, blurred vision, nausea, Dimness of vision, and fainting^(43)^.

SAH has been documented as an important health problem among incarcerated women, with a prevalence of approximately 24%, similar to that found in the general population. However, this condition is mainly present among young women (33 years old on average)^(3,44)^, showing an illness profile similar to that of older women in the general population.

Incarcerated women face high social and health vulnerability, which leads to an accumulation of several risk factors and multiple health disparities^(44)^. Most of the risk factors described in the MRT developed here are modifiable factors that can be controlled through changes in lifestyle and behavior^(41)^.

Excess body weight (overweight or obesity) and a sedentary lifestyle are risk factors addressed by most articles composing this MRT^(2,14-25,27-30,36)^. The systematic review shows that women gained weight in prison more frequently than men, and such gain was associated with SAH^(28)^. A sedentary lifestyle contributes to weight gain, and prisons offer restricted opportunities to exercise in addition to physically limited spaces^(4)^ and high-calorie and processed foods^(2,17,28)^.

Note that a direct relationship exists between overweight, a sedentary lifestyle, and blood pressure levels. The more sedentary an individual is, the more his/her weight gain, and consequently, the greater his/her chances of elevated blood pressure and hypertension. Insufficient physical activity is a prevalent problem worldwide, especially among women^(41)^. Therefore, overweight and a sedentary lifestyle are considered risk factors that deserve special attention from the nursing team, as nurses can promote specific educational actions in female prisons to encourage exercise, a healthy diet, and body weight control^(23)^.

Regarding diet, studies show that the meals provided to incarcerated women are generally rich in sodium and calories^(15,18,29-32)^. This dietary profile contributes to a gradual increase in blood pressure and the development of dyslipidemia, another risk factor related to SAH^(2,14-17,20-21,23-24,27)^. High cholesterol levels contribute to the development of SAH by activating the renin-angiotensin system, decreasing the availability of nitric oxide and endothelial dysfunction^(45)^.

Smoking is another frequent habit among incarcerated women and is considerably prevalent in this population^(2,14-19,21-22,24,33)^. The relationship between smoking and increased blood pressure occurs through the activation of the sympathetic nervous system, which causes an increase in heart rate, blood pressure levels, and myocardial contractility^(46)^.

The harmful consumption of alcohol and illicit substances is also reported as related to blood pressure^(14-15,17-19,22-25,27,35-37)^. Concerning alcohol consumption, there is a higher prevalence of increased blood pressure levels among people who consume more than the daily recommendations. As for the consumption of illicit substances, the effects on blood pressure can be diverse, depending on the type of substance^(41)^; high rates of illicit substance use are verified among incarcerated women^(1)^.

Another important factor is stress^(2,15-16,23-24)^. A prison’s environment may trigger continuous stress due to confinement in a physically limited space with high noise levels and having to deal with threatening interactions with other inmates and guards. These situations may contribute to increased blood pressure levels due to the physiological processes of stress^(2)^. Insomnia is another factor found in prisons that is also related to elevated blood pressure among incarcerated women^(19)^ due to an increased activity of the sympathetic nervous system^(47)^.

The articles comprising the MRT developed here found insufficient knowledge/awareness about risk factors to be relevant to increased risk for imbalanced blood pressure pattern in incarcerated women^(16,23)^. Individuals with poor knowledge and awareness are more likely to adopt a lifestyle detrimental to blood pressure^(48)^.

Educational interventions can improve health in prison institutions as they improve knowledge about risk factors, promote health empowerment, behavioral change, and cardiovascular prevention and control of blood pressure and its risk factors^(5,31)^. Health education actions conducted by nurses based on identifying NDs can potentially improve individuals’ knowledge^(49)^.

The conditions associated with the risk for imbalanced blood pressure pattern among incarcerated women include diabetes^(19,26)^, cardiovascular disease^(14,16,19-20,25)^, and post-traumatic stress disorder^(16)^. Nursing actions cannot independently modify any of these conditions^(6)^. Nonetheless, these must be taken into account in the therapeutic plan of those with ND Risk for imbalanced blood pressure pattern.

The literature shows that populations at risk for imbalanced blood pressure pattern include individuals with a family history of hypertension^(19)^. Genetic factors are known to be related to blood pressure levels^(41)^.

Individuals experiencing social vulnerability, social isolation, and belonging to vulnerable ethnic minorities, e.g., Afro-descendants and those of mixed race, with low socioeconomic and educational levels, and with precarious access to health services, are considered populations at risk for imbalanced blood pressure pattern^(16,24)^. These conditions are classified as psychosocial factors that increase the risk of high blood pressure^(50)^.

Causal relationships and evidence for practice constitute essential elements of an MRT developed to support the theoretical-causal validity of an ND, as evidence supports diagnostic reasoning in nursing, strengthening nurses’ clinical judgment in the nursing process^(9)^. Thus, the entire diagnostic structure is based on evidence to be used in practice.

### Study limitations

This study’s limitation concerns the fact that the theoretical, conceptual model presented here may have restricted the causal relationships between the concepts because it addresses a peculiar population: incarcerated women.

### Contributions to the Nursing field

The MRT of Risk for imbalanced blood pressure pattern allowed to contribute to the phenomenon theoretically and proved necessary as evidence to update the NANDA-I taxonomy, to support the clinical judgment and diagnostic reasoning of nurses and, consequently, contribute to planning actions promoting the cardiovascular health of incarcerated women, and prevent problems related to imbalanced blood pressure patterns.

## CONCLUSIONS

This MRT allowed greater understanding of the causal relationships of the phenomenon Risk for imbalanced blood pressure pattern, enabling the creation of a diagnostic structure with new elements (definition, risk factors, associated conditions, and populations at risk) based on the identification of two essential attributes and 20 antecedents, a pictorial diagram, and nine theoretical propositions.

These causal relationships are expected to be found in the female incarcerated population through other stages of the ND validity process (content validity and clinical validity), which can support the production of evidence and understanding of the phenomenon, favoring more robust evidence of the diagnosis addressed here.
