# Supplementary material for: Risk for imbalanced blood pressure pattern among incarcerated women: Middle-Range Theory
Source: Rev Bras Enferm. 2024 Jul 15;77(Suppl 3):e20230288. doi: 10.1590/0034-7167-2023-0288 (PMC11253836; doi:10.1590/0034-7167-2023-0288)
Supplement: Translation

# Translation: Risco de padrão pressórico desequilibrado em mulheres encarceradas: Teoria de Médio Alcance

**DOI:** 10.1590/0034-7167-2023-0288pt

**Authors:** Gabrielle Pessôa da Silva, Camila Takáo Lopes, Marcos Venícios de Oliveira Lopes, Ryanne Carolynne Marques Gomes Mendes, Jaqueline Galdino Albuquerque Perrelli, Lívia Maia Pascoal, Suzana de Oliveira Mangueira, Francisca Márcia Pereira Linhares

**Affiliations:** I Universidade Federal de Pernambuco. Recife, Pernambuco, Brasil; II Universidade Federal de São Paulo. São Paulo, São Paulo, Brasil; III Universidade Federal do Ceará. Fortaleza, Ceará, Brasil; IV Universidade Federal do Maranhão. São Luís, Maranhão, Brasil

**Keywords:** Diagnóstico de Enfermagem, Teoria de Enfermagem, Pressão Arterial, Mulheres, Prisões

## Abstract

**Objetivos:**

desenvolver Teoria de Médio Alcance para o Risco de padrão pressórico desequilibrado em mulheres encarceradas.

**Métodos:**

estudo de desenvolvimento teórico para a validade teórico-causal do Diagnóstico de Enfermagem Risco de pressão arterial instável. Construiu-se Teoria de Médio Alcance a partir de seis etapas: definição da abordagem para construção da teoria; definição dos modelos conceituais a serem analisados; definição dos conceitos principais; desenvolvimento do diagrama pictórico; construção das proposições; estabelecimento das relações causais e evidências para a prática.

**Resultados:**

identificaram-se dois atributos e 20 antecedentes relacionados ao padrão pressórico desequilibrado; além da construção de um diagrama pictorial e nove proposições teóricas.

**Conclusões:**

a teoria desenvolvida favorece o raciocínio diagnóstico dos enfermeiros e contribui para o planejamento de ações promotoras da saúde cardiovascular das mulheres encarceradas. Ademais, foi possível estruturar uma nova proposta para o diagnóstico Risco de pressão arterial instável, com novo título, nova definição e novos fatores etiológicos.

## INTRODUÇÃO

No contexto do encarceramento, o tabagismo, o uso de álcool e drogas ilícitas^(1)^, as aglomerações, os ruídos e as relações conflituosas e ameaçadoras promovem um ambiente estressante, o que contribui para o aumento da pressão arterial, bem como para o aumento de casos de hipertensão arterial sistêmica após a libertação da prisão^(2)^.

No Brasil, há uma prevalência de 24,2% mulheres encarceradas hipertensas, sendo que essa condição se apresenta majoritariamente em mulheres jovens (média de 33 anos)^(3)^. Mulheres encarceradas geralmente são mais afetadas do que homens e possuem dificuldades para manter os níveis tensionais normais, devido à dieta inadequada e ao sedentarismo, mediante as limitadas oportunidades de realização de atividades físicas dentro da instituição penal^(4)^.

Os enfermeiros que prestam assistência em instituições penais devem identificar os fatores de risco para alteração da pressão arterial, com a finalidade de prevenir a ocorrência de eventos cardiovasculares^(5)^. A identificação desses fatores pode ser facilitada por meio dos Diagnósticos de Enfermagem (DE) da NANDA *International* (NANDA-I). Por outro lado, inconsistências na estrutura diagnóstica podem comprometer a identificação e a nomeação do fenômeno^(6)^.

O fenômeno risco de pressão arterial instável é um DE da NANDA-I definido como “suscetibilidade a forças flutuantes de sangue fluindo através dos vasos arteriais, que podem comprometer a saúde”^(6)^. Essa definição não é clara, o que pode prejudicar o raciocínio diagnóstico e o julgamento clínico do enfermeiro. Além disso, na NANDA-I, tal DE possui apenas dois fatores de risco - inconsistência com o regime medicamentoso e ortostasia, o que pode afetar o reconhecimento do fenômeno pelos enfermeiros.

Espera-se que os estudos apoiem as evidências de validade de um DE, de modo a permitir a expansão de seu uso em diferentes populações. Nesse contexto, a Teoria de Médio Alcance (TMA) é a abordagem metodológica preferida para o processo de validade teórico-causal de um diagnóstico^(7)^.

Como o DE risco de pressão arterial instável necessita de uma definição clara e de mais fatores de risco que permitam sua identificação precisa, recomenda-se a sua validade teórico-causal por meio do desenvolvimento de TMA. Destaca-se que não foram encontrados na literatura científica estudos voltados à construção de teoria de enfermagem para este fenômeno, sendo necessário o desenvolvimento deste estudo, o qual pode contribuir para a operacionalização do Processo de Enfermagem na assistência às mulheres encarceradas. Assim, considera-se este estudo inovador para o campo disciplinar da enfermagem.

## OBJETIVOS

Desenvolver TMA para o Risco de padrão pressórico desequilibrado em mulheres encarceradas.

## MÉTODOS

### Aspectos éticos

Por tratar-se de estudo de desenvolvimento teórico, não necessitou de aprovação do Comitê de Ética em Pesquisa, uma vez que não houve a participação de seres humanos de forma direta.

### Tipo de estudo

Trata-se de estudo de desenvolvimento teórico, orientado para a validade teórico-causal do DE Risco de pressão arterial instável^(7)^. Para isso, desenvolveu-se uma TMA, a qual foi construída a partir de revisão integrativa de literatura e dos conceitos do Modelo de Adaptação de Roy (MAR)^(8)^.

A construção da teoria foi embasada nas seis etapas propostas por Lopes, Silva e Herdman (2017), as quais reiteram o referencial de Roy (2014): definição da abordagem para construção da teoria; definição dos modelos conceituais a serem analisados; definição dos conceitos principais; desenvolvimento do diagrama pictórico; construção das proposições; estabelecimento das relações causais e evidências para a prática^(9-10)^. Ao fim do desenvolvimento teórico, foi possível estabelecer uma proposta de refinamento do DE estudado, o qual assumiu um novo título, que deu o nome à TMA desenvolvida.

### Definição da abordagem para construção da Teoria de Médio Alcance e definição dos modelos conceituais a serem analisados

As abordagens para desenvolvimento da TMA foram: a revisão integrativa da literatura e os conceitos do MAR^(8)^. Esse modelo foi escolhido por considerar que as mudanças vivenciadas pelas mulheres encarceradas ao adentrar na prisão interferem diretamente em diversos aspectos da saúde, inclusive na saúde cardiovascular, por tratar-se de um ambiente de superlotação, com alto nível de estresse e acesso precário ao sistema de saúde, além de poucas oportunidades de modificação de estilo de vida^(1)^. Essas características podem ocasionar adaptações no modo de vida e repercussões importantes para a pressão arterial dessas mulheres.

Os conceitos de Roy utilizados são os de estímulos focais, contextuais e residuais^(8)^. O estímulo focal é aquele mais próximo dos indivíduos, capaz de enfrentá-los e causar maior impacto relacionado à mudança. Estímulos contextuais internos e externos incluem aqueles que influenciam o estímulo focal. Estímulos residuais são aqueles que têm efeitos não centrais^(8)^. Esses estímulos devem ser os antecedentes clínicos (fatores de risco, condições associadas e populações de risco) do padrão pressórico desequilibrado. Para este estudo, o problema adaptativo é o padrão pressórico desequilibrado, o qual foi considerado como um possível comportamento das mulheres encarceradas devido aos antecedentes clínicos/estímulos identificados no estudo. Segundo o MAR, esse comportamento é classificado no modo fisiológico^(8)^.

### Definição dos principais conceitos

Os principais conceitos utilizados foram os atributos essenciais e os antecedentes clínicos^(9)^ do risco de padrão pressórico desequilibrado. Os atributos essenciais são as características do núcleo conceitual do DE. Os antecedentes clínicos para diagnósticos de risco podem ser fatores de risco (elementos que aumentam a suscetibilidade para o diagnóstico e são passíveis de intervenções autônomas de enfermagem), populações em risco (características demográficas não modificáveis e/ou histórico de saúde) e condições associadas (diagnósticos médicos, dispositivos médicos e/ou medicamentos)^(6,9)^. Esses conceitos foram identificados por meio de uma revisão integrativa da literatura, de acordo com as etapas: identificação do problema, busca na literatura, avaliação dos dados, análise dos dados e apresentação dos resultados^(11)^.

### Identificação de problema

O problema foi operacionalizado por meio da estratégia PICo - P (População): mulheres encarceradas; I (Interesse): atributos essenciais e antecedentes clínicos; e Co (Contexto): encarceramento feminino. Assim, obteve-se a seguinte pergunta de pesquisa: “Quais são os atributos essenciais e antecedentes clínicos do risco de padrão pressórico desequilibrado em mulheres encarceradas?”.

### Busca na literatura

A pesquisa bibliográfica foi realizada em maio de 2021, usando os termos MeSH “*arterial pressure*”, “*prisons*”, “*prisioners*”, “*risk factors*”, “*women*” nas bases *Scopus*, CINAHL, Medline/PubMed, *Web of Science*, Embase, *Science Direct* e Cochrane. Para a base de dados LILACS, foram utilizados os Descritores em Ciências da Saúde (DeCS) “pressão arterial”, “hipertensão”, “prisões” e “mulheres” nos idiomas inglês, português e espanhol. Não foram aplicados limites de tempo ou idioma. As estratégias de busca utilizadas nas bases de dados são apresentadas a seguir:

1) *Scopus*, CINAHL, Medline/PubMed, *Web of Science*, Embase, *Science Direct* e Cochrane: “*arterial pressure*” *AND* (*prisons OR prisioners*) *AND* “*risk factors*” *AND women*

2) LILACS: #1) *Hypertension AND Women AND Prisons*; #2) “*Arterial pressure*” *AND Women AND Prisons*; #3) Hipertensão *AND* mulheres *AND* prisões; #4) Pressão arterial *AND* Mulheres *AND* Prisões; #5) *Hipertensión AND Mujeres AND Prisón*

### Avaliação de dados

Os títulos e resumos dos estudos foram lidos de forma independente por dois pesquisadores, seguidos da leitura dos textos completos, obtendo-se os seguintes dados: autores, ano de publicação, delineamento do estudo, local, atributos essenciais e antecedentes clínicos do padrão pressórico desequilibrado. Os critérios de inclusão foram: estudos observacionais primários ou revisões de literatura que definissem o fenômeno de estudo e/ou descrevessem fatores que contribuíssem para o padrão pressórico desequilibrado em mulheres encarceradas. Os critérios de exclusão foram: editoriais, anais de eventos e cartas ao editor.

### Análise de dados

O nível de evidência dos estudos foi avaliado de acordo com a classificação: Nível 1: estudos experimentais; Nível 2: estudos quase-experimentais; Nível 3: analítico observacional; Nível 4: estudos descritivos observacionais; e Nível 5: opiniões de especialistas e pesquisa de bancada. Estudos metodológicos, qualitativos, analíticos transversais ou revisões de literatura que não fossem sistemáticos foram classificados no nível 5^(12)^.

### Apresentação de resultados

Os resultados foram categorizados como atributos essenciais ou antecedentes clínicos. Os antecedentes clínicos foram categorizados como estímulos focais, contextuais ou residuais, a partir dos pressupostos teóricos de Roy (2009) sobre as conceituações do MAR^(8)^, das relações causais evidenciadas na literatura e das reflexões dos autores conforme suas experiências práticas na temática.

Identificaram-se 2026 estudos por meio das buscas, os quais após remoção dos duplicados, totalizaram 755 publicações que foram selecionadas para leitura de títulos e resumos. Dessas, 36 foram lidas na íntegra, sendo 25 incluídas na amostra final. Os dados dos estudos selecionados estão sumarizados na Quadro 1.

**Quadro 1:** Estudos incluídos na Revisão de Literatura para construção da Teoria de Médio Alcance do Risco de padrão pressórico desequilibrado em mulheres encarceradas, Recife, Pernambuco, Brasil, 2023

Autores, ano, local	Desenho do estudo e nível de evidência	Nº de atributos extraídos	Nº de AC extraídos
Bondolfi et al., 2020^(2)^ Reino Unido	Revisão sistemática Nível 1	1	7
Nolan e Stewart, 2017^(14)^ Canadá	Estudo transversal Nível 4	1	8
Agyapong et al., 2017^(15)^ Reino Unido	Revisão sistemática Nível 1	1	9
Arries e Maposa, 2013^(16)^ Estados Unidos	Revisão integrativa Nível 5	2	11
Bautista-Arredondo et al., 2015^(17)^ México	Estudo transversal Nível 4	2	8
Galvão et al., 2019^(18)^ Brasil	Estudo transversal Nível 4	1	7
Hachbardt et al., 2020^(19)^ Brasil	Estudo transversal Nível 4	1	12
Khavjou et al., 2017^(20)^ Estados Unidos	Estudo transversal Nível 4	1	9
Lagarrigue et al., 2017^(21)^ França	Estudo transversal Nível 4	1	6
Plugge et al., 2009^(22)^ Inglaterra	Estudo descritivo prospectivo Nível 4	1	8
Silva et al., 2021^(23)^ Brasil	Estudo metodológico Nível 5	1	11
Vera-Remartínez et al., 2018^(24)^ Espanha	Estudo transversal Nível 4	1	11
Wildeman et al., 2013^(25)^ Estados Unidos	Estudo transversal Nível 4	1	8
Udo, 2019^(26)^ Estados Unidos	Estudo transversal Nível 4	1	9
Nara e Igarashi, 1998^(27)^ Japão	Estudo transversal Nível 4	1	5
Gebremariam et al., 2018^(28)^ Reino Unido	Revisão sistemática Nível 1	--	3
Herbert et al., 2012^(29)^ Reino Unido	Revisão sistemática Nível 1	--	3
Nucci et al., 2019^(30)^ Itália	Estudo metodológico Nível 5	--	4
Mohan et al., 2018^(31)^ Holanda	Revisão Sistemática Nível 1	--	4
Wangmo et al., 2018^(32)^ Suíça	Estudo qualitativo Nível 5	--	2
Ritter et al., 2011^(33)^ Estados Unidos	Revisão sistemática Nível 1	--	1
Valera et al., 2016^(34)^ Nova Iorque	Estudo qualitativo Nível 5	--	1
Gilles et al., 2008^(35)^ Austrália	Estudo transversal Nível 4	--	3
Johnson et al., 2019^(36)^ Canadá	Estudo de coorte Nível 3	--	5
Fazel e Baillargeon, 2011^(37)^ Reino Unido	Revisão integrativa Nível 5	--	3

*AC - Antecedentes Clínicos.*

### Desenvolvimento do diagrama pictórico, formulação das proposições e estabelecimento das relações causais e evidências para a prática

Um resumo gráfico das inter-relações entre os conceitos foi projetado para resumir os elementos da TMA. As proposições foram construídas a partir de afirmações claras que demonstrassem a relação entre os antecedentes clínicos e o diagnóstico, destacando suas especificidades. As relações causais entre os antecedentes clínicos e o padrão pressórico desequilibrado foram identificadas e descritas a partir dos estudos da revisão integrativa e também da consulta a outros artigos científicos, quando esses não apresentavam tais relações. Esta última etapa apresenta as relações clínicas que conduzem ao raciocínio diagnóstico em enfermagem^(9)^.

Com base nos atributos essenciais, nos antecedentes identificados na revisão da literatura e nas relações causais estabelecidas na TMA, realizou-se uma proposta de refinamento do título, definição e fatores etiológicos do DE Risco de pressão arterial instável, considerando o que está previsto na norma ISO 18104 sobre as terminologias de referência em enfermagem^(13)^. Consideraram-se as definições da NANDA-I referentes ao título diagnóstico, sendo o status diagnóstico (diagnóstico de Risco) associado ao eixo foco e ao eixo julgamento. A definição foi construída para fornecer uma descrição clara e precisa do título diagnóstico. Foram considerados fatores de risco como antecedentes que aumentam a suscetibilidade de um indivíduo a uma resposta humana indesejável; as condições associadas estão relacionadas a diagnósticos/procedimentos/dispositivos médicos, e as populações em risco, são grupos de indivíduos com maior suscetibilidade ao fenômeno^(6)^.

## RESULTADOS

### Conceitos principais

Identificaram-se, a partir dos estudos selecionados na revisão integrativa, como conceitos principais, dois atributos essenciais, que foram a “elevação recorrente da força do sangue contra a parede arterial acima do nível desejado”^(2,14-26)^ e a “redução recorrente da força do sangue contra a parede arterial abaixo do nível desejado”^(16-17,27)^. Também foram identificados 20 antecedentes clínicos que estão relacionados ao padrão pressórico desequilibrado, os quais foram categorizados conforme o MAR e estão descritos no Quadro 2.

**Quadro 2:** Categorização dos antecedentes clínicos do Risco de padrão pressórico desequilibrado em mulheres encarceradas, conforme o Modelo de Adaptação de Roy, em estímulos focais, contextuais e residuais, Recife, Pernambuco, Brasil, 2023

Categorização dos estímulos	Definições dos estímulos	Antecedentes clínicos
Estímulos focais	Influenciam diretamente a mulher encarcerada e são os principais responsáveis por aumentar a suscetibilidade dessa mulher para desenvolver o padrão pressórico desequilibrado^(8)^.	Sedentarismo^(2,15-24,27-31)^ Alimentação hipercalórica^(15-16,18-20,22-24,28-31)^ Alimentação hipersódica^(2,15,18-20,22-24,28-32)^ Tabagismo^(2,14-27,30-31,33-36)^ Uso nocivo de substâncias ilícitas^(13,15,17,25,27,35-37)^ Uso nocivo de álcool^(14-15,18-19,22-23,25-27,35-37)^ Ansiedade^(21,23,25)^ Estresse^(2,15-16,19,23,26)^ Transtorno de Estresse Pós-Traumático^(16)^ Síndrome metabólica^(16,21,24)^
Estímulos contextuais	Influenciam de forma indireta as mulheres encarceradas a desenvolverem o padrão pressórico desequilibrado^(8)^.	Insônia^(15,19)^ Excesso de peso corporal^(2,14-30)^ Dislipidemia^(2,14-17,20-21,23-24,26-27)^ Doença cardiovascular^(14,16,19-20,25-26)^ Diabetes^(19,26)^
Estímulos residuais	Estimulam de forma menos intensa o comportamento do padrão pressórico desequilibrado nas mulheres encarceradas. Não está totalmente esclarecida qual a influência desses estímulos para que ocorra um padrão pressórico desequilibrado^(8)^.	Conhecimento/compreensão insuficiente dos fatores de risco^(16,23)^ Indivíduos com histórico familiar de hipertensão arterial^(19)^ Indivíduos em vulnerabilidade social^(2,16-20,22,24-26,31,36)^ Mulheres^(14,16-17,24,26,31,37)^ Indivíduos com idade maior que 30 anos^(14-20,22,25-26)^

### Diagrama pictórico

O pictograma, apresentado na Figura 1, explica as inter-relações entre os conceitos que envolvem o Risco de padrão pressórico desequilibrado nas mulheres encarceradas. O coração está em destaque para representar o organismo humano (mulher encarcerada). O coração humano pode ser influenciado por diversos estímulos que podem gerar um padrão pressórico desequilibrado (elevação ou redução recorrente da força do sangue contra a parede arterial, acima ou abaixo de níveis desejáveis), que está representado pela saída da artéria aorta. Os círculos tracejados indicam que os estímulos poderão ser reclassificados a depender da intensidade com que afetam as pessoas.

**Figura 1:** Diagrama pictórico do Risco de padrão pressórico desequilibrado, considerando o Modelo de Adaptação de Roy, Recife, Pernambuco, Brasil, 2023

### Proposições da Teoria de Médio Alcance do Risco de padrão pressórico desequilibrado em mulheres encarceradas

Emergiram nove proposições teóricas construídas com a inter-relação entre os conceitos (atributos essenciais e antecedentes clínicos/estímulos) do Risco de padrão pressórico desequilibrado em mulheres encarceradas, com base no MAR, a saber:

1. Os estímulos focais, contextuais e residuais atingem o modo fisiológico das encarceradas e comprometem o funcionamento cardiovascular, aumentando a suscetibilidade para o desenvolvimento do comportamento ineficaz padrão pressórico desequilibrado.
2. Os estímulos focais, contextuais e residuais são intrínsecos ou extrínsecos às mulheres encarceradas, podendo ter sido adquiridos antes do aprisionamento e continuados no ambiente prisional, como também podem ter sido iniciados somente após o encarceramento.
3. Os estímulos focais sedentarismo, alimentação hipercalórica, alimentação hipersódica, tabagismo e uso nocivo de álcool e substâncias ilícitas são hábitos de vida frequentemente adotados pelas mulheres encarceradas que podem afetar diretamente a pressão arterial, gerando o comportamento do padrão pressórico desequilibrado (elevação ou redução).
4. Os estímulos focais ansiedade, estresse e o Transtorno de Estresse Pós-Traumático estão relacionados ao confinamento no ambiente prisional e a experiências traumáticas vivenciadas antes ou durante o aprisionamento. Influenciam diretamente o desenvolvimento do comportamento do padrão pressórico desequilibrado (elevação) nas mulheres encarceradas.
5. A síndrome metabólica é um estímulo focal que pode ocorrer em decorrência do acúmulo de alguns fatores de risco cardiovascular nas mulheres encarceradas e pode favorecer intensamente o comportamento do padrão pressórico desequilibrado (elevação), aumentando a vulnerabilidade dessas.
6. A insônia é um estímulo contextual que potencializa o comportamento do padrão pressórico desequilibrado (elevação) e pode estar relacionado à estrutura física do ambiente prisional, bem como às questões ligadas à saúde mental das mulheres encarceradas.
7. Os estímulos contextuais excesso de peso corporal, dislipidemia, doença cardiovascular e diabetes são condições clínicas que potencializam o comportamento do padrão pressórico desequilibrado (elevação) nas mulheres encarceradas.
8. O conhecimento/compreensão insuficiente dos fatores de risco é um estímulo residual que afeta as ações de autocuidado voltadas para a manutenção de uma boa pressão arterial, por isso, ele pode influenciar indiretamente o comportamento do padrão pressórico desequilibrado (elevação) nas mulheres encarceradas.
9. Os estímulos residuais indivíduos com histórico familiar de hipertensão arterial, indivíduos em vulnerabilidade social, com idade maior que 30 anos e mulheres podem influenciar de forma menos intensa o desenvolvimento do comportamento do padrão pressórico desequilibrado (elevação).

### Proposta do diagnóstico de enfermagem Risco de pressão arterial instável

A partir da TMA construída e de seus elementos, foi possível apresentar uma nova proposta para o DE Risco de pressão arterial instável, no intuito de contribuir para a clarificação e o refinamento de sua estrutura. Essa proposta é consequência do processo de validade teórico-causal do DE Risco de pressão arterial instável realizado por meio desta TMA. A seguir, tem-se a estrutura:

Título: Risco de padrão pressórico desequilibrado; Definição: suscetibilidade à elevação ou redução recorrentes da força exercida pelo sangue na parede das artérias, acima ou abaixo dos níveis desejados, o que pode comprometer a saúde; Fatores de risco: conhecimento/compreensão insuficiente dos fatores de risco, excesso de peso corporal, sedentarismo, alimentação hipercalórica, alimentação hipersódica, síndrome metabólica, tabagismo, uso nocivo de substâncias ilícitas, uso nocivo de álcool, ansiedade, estresse, insônia; Condições associadas: dislipidemia, diabetes, Transtorno de Estresse Pós-Traumático, doença cardiovascular; Populações em risco: indivíduos com histórico familiar de hipertensão arterial, indivíduos em vulnerabilidade social, mulheres, indivíduos com idade maior que 30 anos.

A mudança do título foi proposta para representar com maior clareza os atributos essenciais do fenômeno em estudo, de forma a facilitar o reconhecimento do DE pelos enfermeiros durante o processo de julgamento clínico e raciocínio diagnóstico. Os atributos essenciais, isto é, “Elevação recorrente da força do sangue contra a parede arterial acima do nível desejado e Redução recorrente da força do sangue contra a parede arterial abaixo do nível desejado” serviram de base para reformulação do título diagnóstico. Assim, os termos “padrão pressórico” e “desequilibrado” foram adotados por retratarem melhor os níveis da pressão arterial que podem comprometer a saúde, visto que a instabilidade é uma característica da pressão arterial, a qual pode ocorrer ao longo de todo o dia, por diversas causas e mecanismos, sem necessariamente causar comprometimento à saúde das pessoas. Já um padrão pressórico desequilibrado que, de maneira recorrente, está elevado ou diminuído acima ou abaixo de níveis desejáveis, pode levar ao comprometimento de saúde dos indivíduos.

Quanto aos fatores de risco, às condições associadas e às populações em risco, observa-se uma diferenciação da proposta da NANDA-I com a inclusão e a ampliação desses. Isso poderia ser explicado pela população específica utilizada para a construção da TMA, embora perceba-se também esses mesmos achados para a população em geral, conforme evidenciado nas relações causais, explicitadas mais adiante na discussão.

## DISCUSSÃO

As TMA devem abarcar conceitos menos abstratos e com uma maior aproximação aos detalhes da prática de enfermagem. Para essa ciência, as teorias devem focar em uma parte específica de determinado fenômeno, buscando incluir uma quantidade mais abreviada de conceitos e proposições, os quais devem estar relacionados com a pesquisa e a prática^(38)^.

Na área da cardiologia, tem-se, recentemente, o desenvolvimento de um estudo de TMA do conhecimento deficiente em indivíduos com insuficiência cardíaca. Essa teoria foi desenvolvida a partir do mesmo referencial adotado neste estudo e apresentou 11 proposições e 4 relações de causalidade que podem possibilitar o direcionamento do julgamento clínico dos enfermeiros em relação ao fenômeno^(39)^.

A TMA do Risco de padrão pressórico desequilibrado foi desenvolvida a partir da investigação desse fenômeno nas mulheres encarceradas, com vistas à elucidação causal desse campo do conhecimento, para orientar o processo de enfermagem e a prática clínica. De acordo com os estudos que compuseram a TMA, a elevação ou a redução recorrente da força do sangue contra a parede arterial, acima ou abaixo de níveis desejáveis, podem ser consideradas atributos essenciais do Risco de padrão pressórico desequilibrado nas mulheres encarceradas^(2,14-25,27)^. Identificou-se que a elevação é o principal fator que pode comprometer a saúde cardiovascular dessas mulheres. Alguns estudos destacam como parâmetro um valor pressórico maior ou igual a 130x85 mmHg^(19,21)^, número próximo ao adotado como referência pela *American Heart Association* em 2018^(40)^, que estabelece que níveis pressóricos maiores ou iguais a 130x80 mmHg devem ser considerados como Hipertensão Arterial Sistêmica (HAS).

Outros estudos^(15,20,22,24)^ adotam como parâmetros de HAS os valores recomendados pelas diretrizes brasileira e europeia de hipertensão arterial, que são aqueles maiores ou iguais a 140x90 mmHg^(40-42)^. Os parâmetros pressóricos estabelecidos mundialmente reforçam a necessidade de adoção de medidas preventivas cada vez mais precoces em relação à pressão arterial elevada.

Os estudos incluídos nesta TMA não mencionam um valor específico para que seja considerada a hipotensão arterial. Entretanto, deve ser considerado o nível pressórico habitual dos indivíduos. Comumente, valores abaixo de 90x60 mmHg são estabelecidos como parâmetro de hipotensão, especialmente quando associados à tontura, visão turva, náusea, escurecimento da vista e desmaios^(43)^.

Para as mulheres encarceradas, a HAS tem sido documentada como um problema de saúde importante, com uma prevalência em torno de 24%, valor semelhante ao da população em geral. Além disso, tem-se que essa condição se apresenta majoritariamente em mulheres jovens (média de 33 anos)^(3,44)^, o que demonstra um perfil de adoecimento semelhante ao das mulheres mais velhas da população geral.

Constata-se uma elevada vulnerabilidade social e de saúde das mulheres encarceradas, o que permite o acúmulo de vários fatores de risco e múltiplas disparidades em saúde^(44)^. Dentre os fatores de risco descritos na TMA, destaca-se o fato de a maioria serem classificados como fatores modificáveis por meio de mudanças no estilo de vida e de comportamento^(41)^.

O excesso de peso corporal (sobrepeso e/ou obesidade), aliado ao sedentarismo, são fatores de risco contemplados na maioria dos artigos que compuseram a TMA^(2,14-25,27-30,36)^. Uma revisão sistemática demonstrou que mais mulheres ganharam peso na prisão em relação aos homens na mesma condição e esse ganho esteve associado à HAS^(28)^. Ademais, o sedentarismo aparece como fator contribuinte para esse ganho de peso no ambiente prisional em virtude de limitadas oportunidades para realizar atividade física e ambiente com espaço limitado^(4)^. Somado a isso, tem-se o consumo de alimentos com alto teor calórico e industrializados, que também contribuem para o aumento de peso^(2,17,28)^.

Existe uma relação direta entre o excesso de peso, o sedentarismo e os níveis de pressão arterial. Quanto maior o sedentarismo, maior o ganho de peso e, consequentemente, maiores são as chances de elevação da pressão arterial e HAS. Mundialmente, a realização insuficiente de atividade física se apresenta como um problema de significativa prevalência, principalmente entre as mulheres^(41)^. Dessa forma, considera-se que o excesso de peso e o sedentarismo são fatores de risco que merecem uma atenção especial da equipe de enfermagem, já que, no meio prisional feminino, os enfermeiros podem promover ações educativas específicas que estimulem a realização de atividades físicas, alimentação saudável e o controle de peso^(23)^.

Em relação à alimentação das mulheres encarceradas, estudos demonstraram que as refeições fornecidas às mulheres são geralmente ricas em sódio e calorias^(15,18,29-32)^. Esse perfil alimentar contribui para a gradativa elevação da pressão arterial dessas mulheres e para o desenvolvimento de dislipidemia, outro fator de risco encontrado que possui relação com a HAS^(2,14-17,20-21,23-24,27)^. Níveis elevados de colesterol colaboram com o desenvolvimento da HAS pela ativação do sistema renina-angiotensina, pela redução da disponibilidade de óxido nítrico e disfunção endotelial^(45)^.

Outro hábito frequente entre as mulheres encarceradas é o tabagismo, fator que apresenta taxas de prevalência consideráveis nessa população^(2,14-19,21-22,24,33)^. A relação entre tabagismo e a elevação da pressão arterial ocorre pela ativação do sistema nervoso simpático, o qual provoca aumento da frequência cardíaca, dos níveis pressóricos e da contratilidade miocárdica^(46)^.

O uso nocivo de álcool e substâncias ilícitas também foi relatado pelos artigos como sendo fatores que têm relação com a pressão arterial^(14-15,17-19,22-25,27,35-37)^. Em relação ao álcool, percebe-se uma maior prevalência de elevação dos níveis pressóricos nas pessoas que consomem acima das recomendações diárias. Já para o consumo das substâncias ilícitas, os efeitos na pressão arterial podem ser diversos, a depender do tipo de substância consumida^(41)^. Entre as mulheres encarceradas, percebe-se altos índices de consumo de substâncias ilícitas^(1)^.

Outro fator importante é o estresse^(2,15-16,23-24)^. A vivência no ambiente prisional pode levar ao desencadeamento de estresse contínuo pelo confinamento em espaço físico limitado, com alto nível de ruídos e presença de interações ameaçadoras com outros detentos e guardas, situações que podem contribuir para a elevação dos níveis de pressão arterial devido aos processos fisiológicos do estresse^(2)^. A insônia também é um fator evidenciado nas prisões e tem relação com a elevação da pressão arterial de mulheres encarceradas^(19)^ devido à atividade aumentada do sistema nervoso simpático^(47)^.

O conhecimento/conscientização insuficiente sobre os fatores de risco foi encontrado nos artigos que compuseram a TMA como relevante para o aumento do risco de padrão pressórico desequilibrado nas mulheres encarceradas^(16,23)^. É provável que indivíduos com baixo nível de conhecimento e conscientização adotem hábitos de vida prejudiciais à pressão arterial^(48)^.

Intervenções educativas podem melhorar a saúde nas instituições prisionais, visto que podem contribuir para o conhecimento dos fatores de risco, empoderamento acerca da saúde, mudança de comportamento, e, consequentemente, a prevenção cardiovascular e o controle da pressão arterial e seus fatores de risco^(5,31)^. Ações de educação em saúde realizadas por enfermeiros com base na identificação de DE têm potencial para aumentar o conhecimento dos indivíduos^(49)^.

Dentre as condições associadas ao risco de padrão pressórico desequilibrado nas mulheres encarceradas, tem-se a diabetes^(19,26)^, a doença cardiovascular^(14,16,19-20,25)^, e o transtorno de estresse pós-traumático^(16)^. Todas essas condições não são passíveis de modificação de forma independente pela enfermagem^(6)^, mas devem ser consideradas no plano terapêutico das pessoas com o DE Risco de padrão pressórico desequilibrado.

Em relação às populações em risco para o padrão pressórico desequilibrado, a literatura demonstra que os indivíduos que possuem histórico familiar de hipertensão^(19)^ têm um risco maior de apresentar desequilíbrio no padrão pressórico. Sabe-se que os fatores genéticos têm relação com os níveis de pressão arterial^(41)^.

Indivíduos que vivem em situação de vulnerabilidade social, em isolamento social, que fazem parte de minorias étnicas vulneráveis - negros e pardos, de baixo nível socioeconômico e educacional e com acesso precário a serviços de saúde são consideradas populações em risco para o padrão pressórico desequilibrado^(16,24)^. Essas condições são classificadas como fatores psicossociais e aumentam o risco para a hipertensão arterial^(50)^.

As relações causais e evidências para a prática constituem-se como elementos importantes de uma TMA construída com a finalidade de apoiar a validade teórico-causal de um DE. Essas evidências auxiliam na condução do raciocínio diagnóstico em enfermagem, o que pode fortalecer o julgamento clínico dos enfermeiros na operacionalização do processo de enfermagem^(9)^. Assim, toda a estrutura diagnóstica apresenta-se fundamentada a partir de evidências para seu uso na prática.

### Limitações do estudo

Como limitação do estudo, enfatiza-se que o modelo teórico conceitual construído pode ter restringido as relações causais entre os conceitos por tratar-se de uma população peculiar, que são as mulheres encarceradas.

### Contribuições para a área da Enfermagem

A TMA do Risco de padrão pressórico desequilibrado permitiu o estabelecimento de um aporte teórico sobre o fenômeno e mostrou-se necessária como evidência para a atualização da taxonomia da NANDA- I, com vistas a favorecer o julgamento clínico e raciocínio diagnóstico dos enfermeiros e, consequentemente, contribuir para o planejamento de ações promotoras da saúde cardiovascular das mulheres encarceradas, bem como para a prevenção de agravos relacionados ao padrão pressórico desequilibrado.

## CONCLUSÕES

A TMA foi desenvolvida e permitiu maior compreensão das relações de causalidade do fenômeno risco de padrão pressórico desequilibrado, sendo possível a criação de uma estrutura diagnóstica com novos elementos (definição, fatores de risco, condições associadas e populações em risco) a partir da identificação de dois atributos essenciais e 20 antecedentes, bem como da construção de um diagrama pictorial e nove proposições teóricas.

Espera-se que essas relações de causalidade possam ser constatadas na população encarcerada feminina também a partir da realização das demais etapas do processo de validade de um DE (validade de conteúdo e validade clínica), as quais podem auxiliar na produção de evidências sobre a compreensão do fenômeno, bem como favorecer o aumento do nível de evidência do diagnóstico estudado.
